# Mapping of colorectal cancer scientific landscape between South Africa and Brazil: a bibliometric analysis

**DOI:** 10.3332/ecancer.2021.1216

**Published:** 2021-04-01

**Authors:** Sphindile Magwaza, Guido Van Hal, Muhammad Hoque

**Affiliations:** 1 Faculty of Medicine and Health Sciences, Department of Social Epidemiology and Health Policy (SEHPO), University of Antwerp, Campus Drie Eiken, Building S University Square 1, 2610 WILRIJK Antwerp, Belgium; 2 Health Systems Trust, 1 Maryvale Rd, Dawncliffe, Westville, Durban 3629, South Africa; 3 Management College of Southern Africa, 26 Samora Machel St, Durban Central, Durban 4001, South Africa

**Keywords:** cancer, colorectal cancer, South Africa, Brazil, bibliometrics, scientific landscape

## Abstract

**Background:**

The five BRICS (Brazil, Russian, Indian, China and South Africa) countries bear a significant proportion of the world’s global cancer burden.

**Aim:**

The aim of this paper is to map the scientific landscape related to colorectal cancer (CRC) research published related to South Africa (SA) and Brazil (BRA).

**Methods:**

We used the bibliometric analysis technique to identify and map the scientific publications on CRC related to SA and BRA. We identified the document type, authors, research organisations, countries, funding sources, most relevant journals, research areas, citation reference counts, journal impact factor (IF) and open access designations in CRC scientific landscape for both countries. We analysed publications from January 2000 to August 2020 as indexed in the Web of Science Core Collection, most covering scientific medical related research and used descriptive statistical data analysis to synthesise the data.

**Findings:**

During the period 2000–2020, there were 80 and 176 peer-reviewed publications on CRC related to SA and BRA, respectively. The majority were original research articles. Sixty-six percent identified had a primary (first) author affiliated to SA research institution and 87% had primary author affiliated to the BRA research institution. Overall, 275 authors published CRC related to SA and 1,025 authors published CRC related to BRA. The leading research organisation in SA was the University of Witwatersrand (Wits, 26%) and was the University of Sao Paulo (23%) for in BRA. The publications, related to both countries, mostly focused on oncology. The South African Medical Journal (10%) produced the most articles for SA with IF = 1.285; and the Value in Health (7%) for BRA with IF = 1.736. The median cited reference count was 32 for SA publications and 34 for BRA publications. There were 49% and 39% of publications without any open access designations for SA and BRA, respectively.

**Conclusions:**

Mapping CRC scientific publications highlighted potential benefits of developing an informed CRC national research plan in each country to promote concerted effort to better understand the risk factors, treatment and advocate for funding as stimulus for increased CRC research outputs that can inform policy development and influence practice to help reduce and control the CRC burden in both countries.

## Introduction

The Global Cancer Observatory (2018) stated that the colorectal cancer (CRC) incidence rate, in South Africa (SA), is 14.4 per 100,000 populations, with the incidence rate of 7.3 and 7.1 per 100,000 for males and females, respectively. It is the 5th most frequent cancer in SA and it is estimated that by year 2030, the number of new cases will increase by 39% while the number of deaths will increase by 40% in 2030 in SA [1].

The CRC age standardised (world) incidence rate (ASIR) estimate is higher in SA than estimates of the Southern African regional ASIR estimate (14.4 versus 13.4 per 100,000) based on the WHO global Cancer Report of 2018 [2]. Similarly, Brazil’s (BRA’s) CRC ASIR is higher than its South American regional ASR estimate (19.6 versus 18. 6 per 100,000), as reported by GLOBOCAN 2018 report. In BRA, according to the GLOBOCAN 2018 report, the CRC mortality ranked fourth among cancer related deaths, with the CRC age-standardised mortality rate 1.2 times higher among Brazilian males compared to females (4.91 and 3.96 per 100,000 populations, respectively) in 2015 [3].

The five BRICS countries (Brazil, Russian, Indian, China and South Africa) account for more than 40% of the global burden of disease [2]. Based on the GLOBOCAN country fact sheets, the five countries collectively have 46,3% of new CRC cases in 2020 (both sexes, all ages). As both countries are ranked among the upper middle income countries, changes in diet and lifestyles have been considered to increase the risk factors for non-communicable diseases (NCDs) [4, 5, 7].

While the European and North American countries have remained the top producers of CRC research, the BRICS, particularly SA and BRA, have also emerged, in recent years, as part of the top 10 producers of CRC research among upper and lower middle income countries as ranked by the World Bank. It has been reported that the average number of CRC research related to SA and BRA has significantly increased since 2009 [1, 7].

The purpose of this study was to map and assess the volume of publications on CRC related to both countries to identify research gaps and advocate for and highlight missed opportunities for expansion of scientific research, funding and partnerships within and between the two countries.

By using this analysis, we were able to identify the number of publications produced in the period of analysis, the year of each publication, the number of authors and their affiliations per publication to review the interaction and collaboration between the two countries and other countries, the types of research organisations, funding agencies supporting CRC research, the types of research focus, types of documents and journals that are sources of publications. We also identified the number of cited reference counts as well as the type of open access designations linked to these publications.

## Materials and methods

We analysed data using bibliometric analysis approach to identify and map the scientific publications on CRC related to SA and BRA countries. Data from scientific publications (articles only) were collected from Thomson Reuters’ Web of Science Core Collection (WoS). The WoS variety of secondary information available for its indexed papers enabling deeper bibliometric analysis [8–9].

In view of the rising number of new cases of CRC in both countries, the paper attempts to describe and identify whether there has also been an increase in scientific publications produced related to CRC in both countries. We used bibliometrics to map the scientific publications [9].

The search was carried out in August 2020 using the advanced search mode of WoS and the following query: (CRC = (Colorectal cancer or C = colon and R = rectal cancers) and cu = (South Africa or Brazil or Brasil or ‘Republic of South Africa’) AND DOCUMENT TYPES: (Article) Indexes = SCI-EXPANDED Timespan = 2000–2020.

The selected CRC descriptors were from the medical subject headings of the National Centre for Biotechnology Information. To exclude articles whose focus was not CRC, the search used the title (ti) field rather than the topic (ts) field. The ts field encompasses articles’ titles, abstracts and keywords (from authors and keywords plus) and abstracts and keywords (from authors and keywords plus). ‘Keywords plus’ is based on WoS editorial readings of the titles of the articles’ bibliographic references, which can create a source of possible ‘garbage’ in the analysis. We restricted our query to only ‘document type’ articles because these are usually more complete and relevant to advanced stages of research [9–10].

We selected the ‘citation indexes’ Science Citation Index Expanded index (SCI-EXPANDED) to narrow the focus on research related to biomedical science to avoid publications indexed in social sciences, arts and humanities (Science Citation Index Expanded). Our period of analysis began in January 2000 as this data is available on the WHO Global Observatory for Cancer which started to publish global CRC data on cancer incidence and mortality during this period [1, 2, 3].

The search collected 80 records of CRC articles related to SA and 176 related to BRA. We imported the raw data from WoS (plain text format) into the Excel 2007 software where we were able to remove duplicates using the validation and remove duplicates command tool and deleted ‘Research Areas’ on topics not related to the aims of this work (such as skin, HIV related cancer, human papillomavirus (HPV), cervical cancer). We used the filter tool on Excel to cluster components for analysis including ‘document type’; ‘year of publication’; ‘Research Area’ indexed in WoS to classify articles by subject (Web of Science Core Collection Research Area). This filtering enabled us to get the total number of articles, manually create tales for further synthesis of data to enable correct interpretation [9–10].

We also included the fields ‘Author Affiliations (Organisation and City and Country)’; addresses and ‘Keywords (authors)’ to verify affiliation. We also used sort (‘highest to lowest’) to identify the highest numbers and manually capture number per category; identify blanks and outliers to clean the data. We used the Excel statistical formula to conduct descriptive statistical data that covered the full period of analysis (2000–2020).

Lastly we searched the Directory of Open Access and the Impact Factor (IF) Database for the scientific journals to determine the IF by publication year [23].

## Results

Between 2000 and 2020, there were 80 and 176 peer-reviewed, scientific articles on CRC published with authors related to SA and BRA, respectively. Of the 80 CRC publications related to SA, 53 (66%) articles had a primary author from South African institution, 12 (15%) had authors with primary affiliation from USA; five (6%) had authors affiliated to Canada and France and four (5%) had authors affiliated to Australia and Belgium.

Of the 176 CRC publications related to BRA, 153 (87%) articles had primary author with affiliation to Brazil institution, 31 (18%) had authors with primary author affiliated to the USA; eleven (6%) had authors affiliated to Canada and 10 (5%) had authors affiliated to England and Spain.

Many publications on CRC and SA included authors from other countries and less authors related to one of the BRICS country. Only three (4%) publications had authors affiliated to China; one affiliated to Russia and one affiliated to India. All publications from SA were written in English. While the majority of publications on BRA were written in English (166 (93%)); however, nine (5%) were written in Portuguese and one (1%) was written in Spanish.

**Figure 1.**
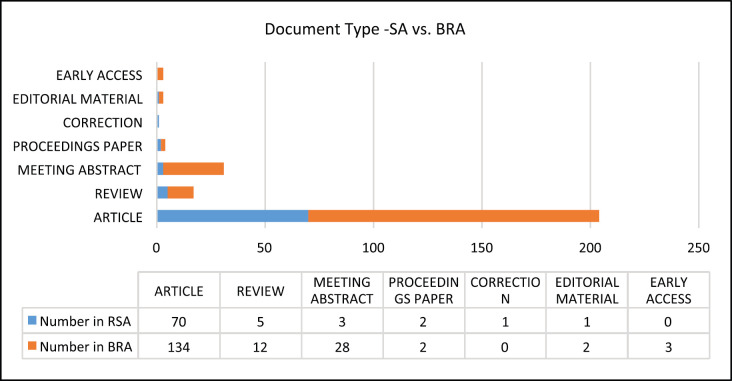
Document type published in SA and BRA: January 2000–August 2020.

The top three document types published on CRC related to SA were articles (70, 88%); followed by reviews (5, 6%) and meeting abstracts (3, 4%). A different sequence for BRA with the first document type being articles (134, 76%); followed by meeting abstracts (12, 16%) and lastly the reviews (1, 7%). As depicted in Figure 1, BRA published 8% more meeting abstracts than were published in SA.

Some authors and co-authors had multiple affiliations. Hence the documents published on CRC that included SA exclusively or SA as part of the world or part of an Africa region had 27 affiliated countries. These countries were located in the North and South America, Americas, Europe; South Asia and the Middle East. Noting that the identified publications accessed via WoS were less than 100, on CRC related to SA, we therefore considered and captured all listed author affiliations.

When analysing the publications by authors, we noted that there were more publications on CRC published with authors related to BRA than those related to SA. The vast majority of authors only produced one publication between the period of analysis. In total, there were 275 authors that had published CRC related to SA. Whereas, for BRA, there were 1,025 authors.

Table 1 outlines the top four authors and the number of publications produced on CRC related to SA and BRA by each author. Only one author was included in eight (10%) publications on CRC related to SA. Three authors were included in five (6%) publications; four authors were included in four (5%) publications and four authors were included in three (4%) publications.

Only one author was included in ten (6%) publications on CRC related to BRA. Four authors were included in eight (5%) publications; two authors were included in seven (4%) publications and five authors were included in six (3%) publications.

The publication year was analysed to determine which year had the most publications on CRC related to SA and BRA. For SA, the number of publications ranged between 1 and 11, while for BRA the number of publications ranged between 1 and 17 during the period of analysis, with 17 published in 2015. The highest number of publications on CRC related to SA was in 2018, with eleven papers published. The highest number of publications on CRC related to BRA was in 2015, with 17 publications. Over 3 years, from 2015 to 2018, BRA produced the highest number of publications (*n* = 62) related to CRC.

**Figure 2.**
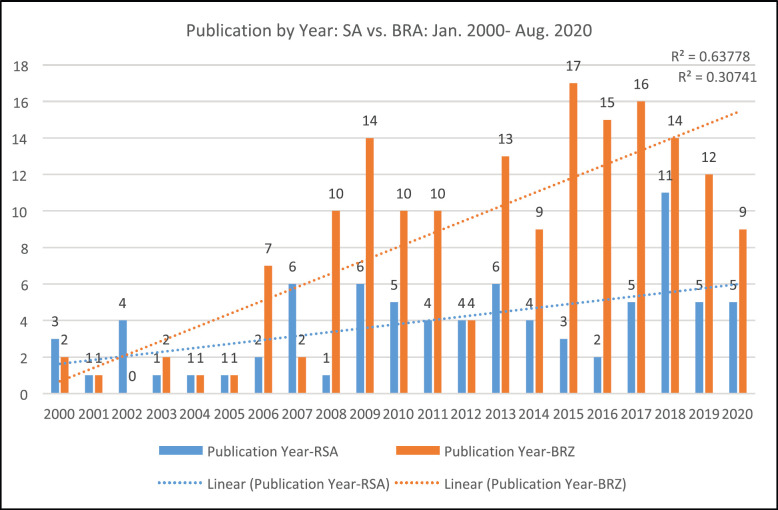
The frequency of publications by year on CRC in SA and BRA: Jan. 2000–Aug. 2020.

The majority of authors produced one CRC article related to SA and BRA, respectively, during the study period. Overall, as per Figure 2, there were more publications on CRC produced related to BRA than those related to SA. Furthermore, the strength of association between the year of publication and the number of publications related to SA is at *r* = 0.55 as compared to an association at *r* = 0.81 for publications on CRC related to BRA.

In BRA, total number of publications increased from 2006, 1 year earlier than SA and produced more publications than SA from 2008 until 2020. SA only produced more publications than BRA in the year 2000, 2002 and 2007. Although a steady increase in the number of publications relayed to BRA, from 2008, however a slight decline was observed over 3 years since 2017. However, for the year 2020, only publications produced within 8 months of 2020 were analysed.

We also analysed at the research organisations affiliated (Table 2a and b) to the authors that have published articles on CRC related to SA and BRA. The top three research organisations affiliated to the publications related to SA were the University of Witwatersrand (Wits – 26%), Cape Town (UCT – 19%) and KwaZulu-Natal (UKZN – 10%), respectively.

Similarly, in BRA, the first three research organisations were University of Sao Paulo (23%), A C Camargo Cancer Centre (10%) and Hospital de Cancer De Barretos (8%).

**Figure 3.**
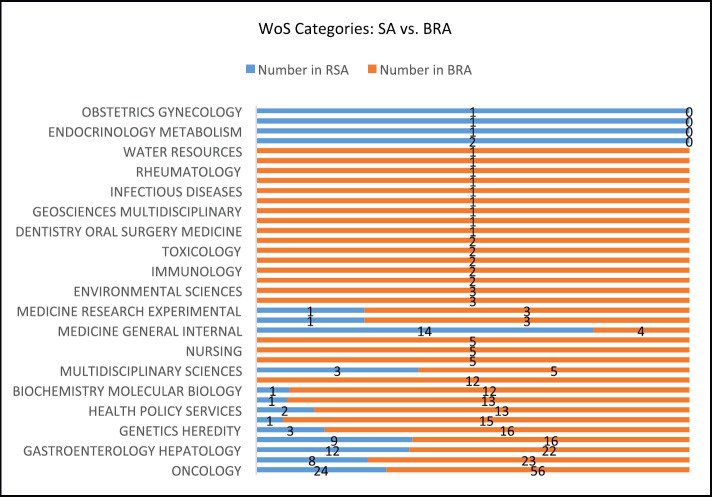
Web of Science research categories for CRC related to SA and BRA: Documents published between Jan. 2000–Aug. 2020.

Reviewing the type of research categories on CRC publications related to SA within the period, we identified oncology (24, 30%); medicine (14, 18%); gastroenterology and hepatology (12, 15%); surgery (9, 11%); and public health, environmental and occupational health (8, 10%) as the top five research categories. There were more research categories indexed on CRC publications related to BRA than those related to SA (Figure 3). The top five research categories identified related to BRA were oncology (56, 32%); public health, environmental and occupational health (23, 13%); gastroenterology and hepatology (22, 13%); Surgery (16, 9%) and Genetic and Heredity (16, 9%); and Nutrition (15, 9%).

When analysing the funding agencies, the Medical Research Council of South Africa, Welcome Trust through the Southern African Consortium for Research Excellence Initiative and the Canadian Institute of Health Research were top three funding agencies declared on the CRC publications related to SA.

**Figure 4a and b.**
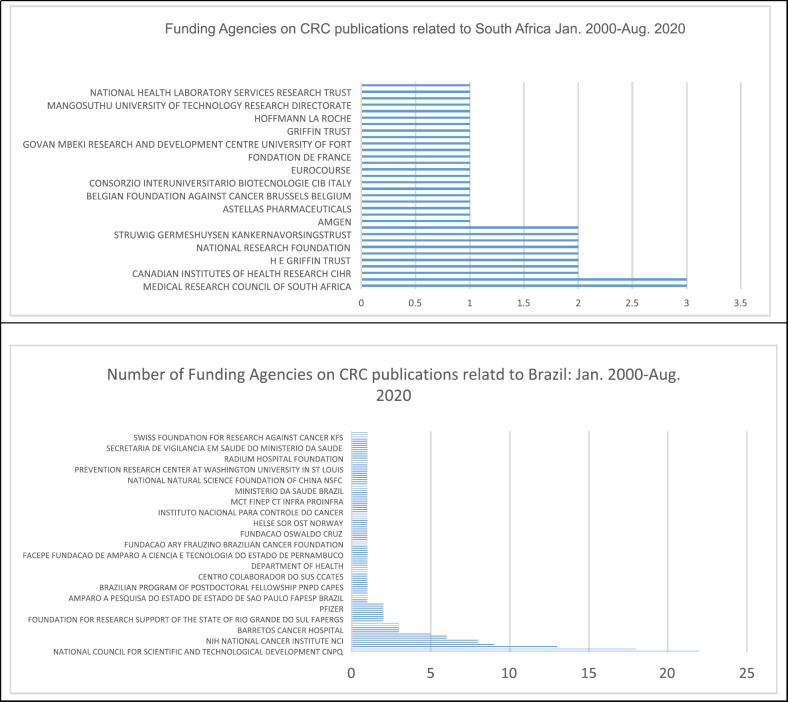
Funding agencies on CRC publications related to SA and BRA: Jan. 2000–Aug. 2020.

The National Council for Scientific and Technological Development and The National Institute for Health (NIH-USA) were the two top funding agencies declared on the CRC published document from BRA, shown in Figure 4a and b.

Twenty-three percent of CRC articles related to SA were published by a local or regional journal, while 28% of the documents on CRC related to BRA were published internationally.

The top four journals that published papers on CRC affiliated to SA were the South African Medical Journal known as SAMJ (10%), South African Journal of Surgery (8%) and International Journal of Cancer (6%). The American Journal of Gastroenterology, Annals of Oncology, MBC complementary and Alternative Medicine, Cancer Epidemiology and European Journal of Cancer and Cancer Prevention all published 3% of CRC articles affiliated to SA.

The top four journals that published papers on CRC affiliated to BRA were: Genetics and Molecular biology (7%), International Brazil Journal of Urology (5%), Diseases of the Colon Rectum (3%), Acta Reumatologica Portuguesa (3%), Annals of Oncology (3%) and Biological Research (3%). The Cadernos De Saude (2%), Ciencia Saude Colectiva, Clinical Oncology, Einstein Sao Paulo, Indian Journal of Pharmacology all published 2% of the papers affiliated to BRA.

When we reviewed the CRC publications by source titles and country, we identified top source titles as depicted in Figure 5a and b). The CRC articles related to SA were published by the South African Medical Journal (SAMJ, 10%), followed by the South African Journal of Surgery (8%) and the International Journal of Cancer (5%).

The Genetics and Molecular Biology Journal (7%) and the International Journal of Urology (5%) were top two journals that published articles on CRC related to BRA.

The CRC publications related to SA and BRA were further analysed to identify common international journals that have published CRC articles related to the two countries. We found 20 journals as outlined in Table 3. However, the International Journal of Cancer (5%), Annals of Oncology (3%), Cancer Epidemiology (3%), the European Journal of Cancer Prevention (3%) and the World Journal of Gastroenterology (3%) were the most common journals for CRC publications related to SA. While, Annals of Oncology (3%) and Disease of the Colon Rectum (3%) were the two most common journals that published CRC articles related to BRA.

**Figure 6.**
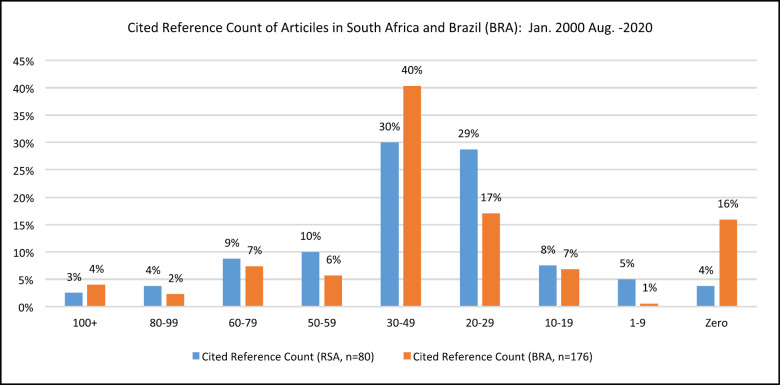
Cited reference count of publications on CRC related to SA and BRA: Jan. 2000–Aug. 2020.

We identified the following similarities and differences when analysing cited reference counts. The cited reference counts were grouped into nine categories, as shown in Figure 6. In both countries, the highest number of publications was in the 30–49 cited reference count category, with 71 (40%) publications related to BRA leading with most counts and SA following with 24 (30%) publications. The second highest number of publications related to both countries was in the 20–29 cited reference count category, with SA leading with most counts with 23 (29%) publications and BRA following with 30 (17%) publications.

The average cited reference count was 38, the mode was 28 and median was 32 for publications on CRC related to SA. The average cited reference count was 36, the mode was 0 and median was 34 for publications on CRC related to BRA. The difference identified between the two countries was in the number of publications that did not have any cited reference count. There were 28 (16%) publications related to BRA and were only three (4%) publications related to SA.

Nonetheless, we also noticed that 4% of publications related to BRA were in the 100+ cited reference count category compared to 3% of publications related to SA in that category. However, the number of publications related to SA was the majority in six of the eight cited reference count categories.

**Figure 7.**
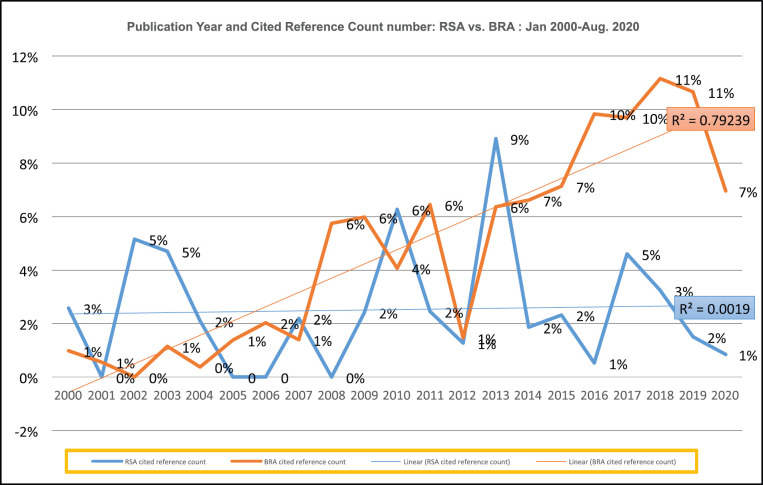
Publication year and cited reference count number: SA versus BRA: Jan. 2000–Aug. 2020.

Figure 7 depicts, the highest number of cited reference count for publications related to SA was reported in the year 2013 with 9%, whereas for BRA was in the years 2018 and 2019 with 11% cited reference counts. SA’s top five highest cited reference counts were in the years 2017 (5%), 2013 (9%), 2010 (6%), 2003 (5%) and 2002 (5%). BRA’s top five highest cited reference counts were in the years 2020 (7%), 2019 (11%), 2018 (11%), 2017 (7%), 2015 (7%) and 2014 (6%).

**Figure 8.**
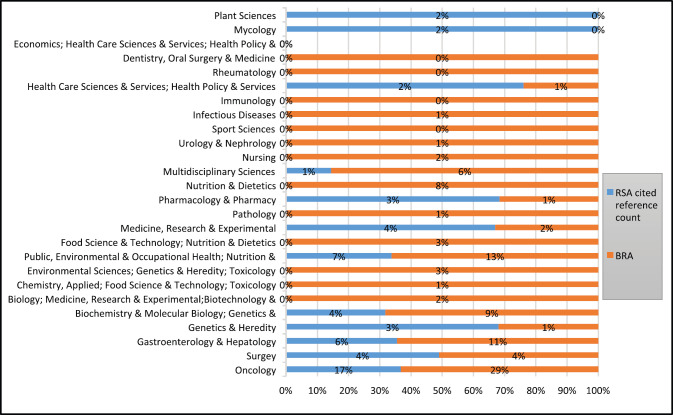
WoS categories and cited reference count number: SA versus BRA: Jan. 2000–Aug. 2020.

The WoS categories with most cited reference counts are depicted by Figure 8, for CRC publications related to SA and BRA (Figure 8). Oncology (17%; 29%); Public, Environmental & Occupational Health (7%;13%); and Nutrition & Dietetics and Gastroenterology & Hepatology category (6%; 11%) were the WoS categories with the most cited reference count for publications related to both countries, respectively.

Medicine, Research & Experimental and Biochemistry & Molecular Biology were at 4% while Genetics & Heredity (3%) was the next WoS category with most cited reference count for publications related to SA. Other WoS categories with cited reference count for publications related to BRA included Biochemistry & Molecular Biology (9%); Nutrition & Dietetics (8%) and Multidisciplinary Sciences (6%).

Publications related to SA that had the most cited reference count were published through local journals, while BRA had most cited reference count papers published through international journals, as outlined in Table 4.

Table 5 outlines the highest number of CRC publications related to SA by journal type and IF, with highest IF highlighted in bold.

Eight articles were published by the SAMJ with the IF = 1.285; six articles were published by the SA Journal of Surgery without any IF; four articles were published by the International Journal of Cancer with the IF = 5.145; two published by the Annals of Oncology with the IF = 18.274 and the American Journals of Gastroenterology with the IF = 10.171.

The majority of journals had published only one article on CRC research related to SA. Among these publications, the top four journals with highest IF were CA-A Cancer Journal for Clinicians (IF = 292.278); British Medical Journal (IF = 30.223); Lancet Oncology (IF = 33.752) and Gastroenterology & Hepatology (IF = 14.789).

Table 6 outlines the highest number of CRC publications related to BRA by journal type and IF, with highest IF highlighted in bold. There were 12 articles published by the Value in Health with the IF = 1.736; nine articles published by the Annals of Oncology with IF = 14.196; five articles published by the Journal of Clinical Oncology with IF = 14.314 and four articles were published by the Diseases of the Colon and Rectum with IF = 3.556.

The majority of journals had published only one article on CRC research related to BRA. The top seven journals with highest IF were Lancet Oncology (IF = 23.296); Clinical Oncology (IF = 18.230); Oncology (IF = 15.389); Journal of National Cancer Institute (IF = 15.271); Lancet Gastroenterology and Hepatology (IF = 14.789); Journal of Clinical Oncology (IF = 14.314) and Annals of Oncology (IF = 14.196).

**Figure 9.**
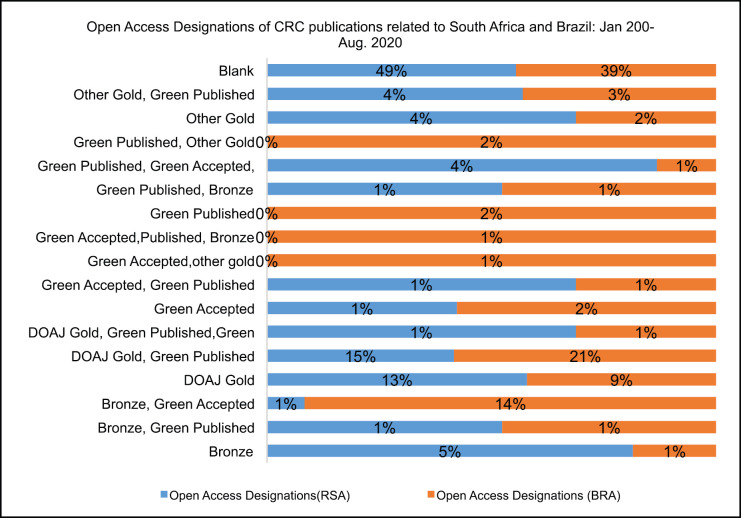
Open access esignations between publications related to SA and BRA: Jan. 200–Aug. 2020.

Noting Figure 9, there were 49% and 39% of publications without any open access designations related to SA and BRA, respectively. About 15% and 21% of publications related to SA and BRA, respectively, had Directory of Open Access Journals (DOAJ) gold, green published, designations. There were more publications with bronze designations related to BRA (16%) than related to SA (7%). There were a considerable number of publications with DOAJ gold designations, 13% and 9% related to SA and BRA, respectively.

## Discussion

This bibliometric study was conducted on CRC publications related to SA and BRA published between January 2000 and August 2020. These are two of the five BRICS countries. The most common type of documents published related to both countries was articles, with BRA leading at 134 published articles indexed in WoS.

The number of publications identified per country indicates that there has been a rapid increase in the production and publications on CRC originating from both countries since the year 2000. A significant rise, in the number of publications, was identified between the years 2006 and 2016, showing a steeper trend line for publications related to BRA than related to SA. The differences in the number of publications by year as noted in Figure 2 could be attributed to the variance in CRC burden between the two countries and differences in CRC surveillance systems implemented in both countries [7, 10–15]. The increase of CRC related articles may be driven by the renewed emphasis on NCDs, mainly Cancer Control by WHO [3] which gained momentum in the following years with scale up of CRC screening programmes in all WHO regions for early detection, treatment and cancer control to reduce morbidity and mortality [10].

During analysis, we also found that between the years 2018 and 2020, there was a decline in the number of production of CRC-related publications for both countries. However, no conclusion can be drawn from this as most scientific articles originating in 2020 may or may not be indexed in the Web of Science Core Collection [8–9]. Nevertheless, the total number of publications indexed by the national bibliometric system during the period of analysis was 176 and 80 for BRA and SA, respectively.

Our findings show that the volume of publications per country may have been influenced by the type of research organisations, affiliation to other authors from different countries, source titles requirements and availability of funding. It also reflects the importance of multi-country collaboration on CRC research.

Our analysis also showed that the most productive organisations in CRC research were a few public organisations: universities or research institutions or teaching hospitals. The top three research organisations affiliated to the publications related to SA tertiary education institutions linked to tertiary or regional hospitals in the three biggest cities in the country. The three cities are known to provide CRC related screening, treatment and control services and interventions [13–15].

In addition, the three research organisations are ranked among top universities in the world, with Wits University ranked as the highest ranked university in Africa by the 2017 Academic Ranking of World Universities. The Quacquarelli Symonds (QS) World University Rankings also ranked UCT as the highest-ranked African, private-public partnership university with the faculty of Medicine rated among the top hundred in the world. The UKZN, a private university, has gained reputation in science, technology, engineering and mathematics world-wide, while leading in the areas of physical sciences and engineering, nationally [16–18].

When reviewing the affiliations, it was evident that there is less interaction within each country and between SA and BRA on CRC research. Competition for limited resources could be both the hindrance affecting within interaction or be the impetus towards stronger in-country collaboration.

We were not surprised to identify better interaction within the region of each country (example: SA and Zimbabwe noting the proximity and regional cooperation) [15], as well as the international collaboration influenced by the former political history of each country, with BRA collaborating with Portuguese or regional countries such as Chile or Uruguay [1–2].

The minimum interaction on CRC research between SA and BRA is noted with concern, bearing in mind that the BRICS partnership is highly regarded as a new global force with greater potential to influence global health [7]. The minimum interaction may have been affected by language differences due to colonial history of both countries, however, both countries are categorised as the middle income countries by the World Bank and the two countries have the highest CRC incidence and mortality rate in this category. Less collaborations may also indicate greater independence of each country, preference and choice of its contribution and unique voice it chooses to express within the global health space [7]. Hence, there is an expectation for the two countries to collaborate and share lessons learned with their regional country members to lower CRC incidence and mortality.

As highlighted by this study, more than 60% of publications indicated receiving funding from international agencies, including the private sector. The three organisations are located in Sao Paulo, the Brazilian capital that has tertiary education institutions leading in teaching, research and linked international recognised hospitals that provide CRC related treatment and services. The University of Sao Paulo, is ranked within top 100 by the Global Employability University Ranking (A global employability ranking, designed by HR consultancy Emerging and published exclusively by Times Higher Education, reveals which universities produced the most employable graduates or are the best at preparing students for the workplace.) and QS Global World Rankings 2021. The A C Camargo Cancer Centre is world acclaim centre that offers an integrated multi-disciplinary approach to diagnosis, treatment, education and research and acts a country tertiary institution [17–19].

The Hospital de Cancer De Barretos offers advanced oncology care funded by the public health systems, a high volume site, with coverage of 27 Brazilian states. Hence, the rankings and reputations may also influence the type of research focus, amount and type of funding these organisations attract (Figure 4a-b) and the number of publications generated and the type of journals chosen for and that accepts manuscripts related to both countries. Noting that the high number of publication that did not include funding sources, reflect, funding gaps limiting CRC research activities, especially in SA, when compared to BRA and other European countries [8].

Evidently, based on Figure 3 above, there are diverse research categories indexed in WoS in both countries. However, publications related to BRA had more variety of categories than those related to SA. Despite the limited number of publications on CRC related to the two countries indexed in WoS, we found that publications generally covered a wide spectrum of areas, but focused mainly on oncology, public environmental health and gastroenterology-hepatology, which are driving factors towards the rising burden of CRC [13–16]. Authors’ interests and expertise are also factors that may influence the research focus and subsequent classification by WoS, allowing for the keywords and the abstract structure of the article.

The international journals were also targeted for publication of the CRC work related to both countries. The international journals were mainly located in Europe or North America, and less so within the regions of the country of origin. The number of authors, authorship sequence and author affiliations could have influenced the type of international journal targeted for publication of CRC related to both countries. We also noted that when the South African authors preferred to publish articles through internal South African or other international journals, that may have affected bibliometric and indexing process as these journals may have aligned to other indexing systems such as used in PubMed or Scopus more than those used in WoS, hence, this may have influenced the type and number of research categories.

There was a moderate association between the year and the number of cited reference counts for publications related to SA. However, the association was strong between the year and number of cited reference counts for publications related to BRA. Equally, some of publications with other international affiliated co-authors may have had better publication opportunities through international journals, which in turn, may likely to be highly cited due to open access designations of these journals.

In this study, no alignment was observed between the type of journal, the frequency of publications and the IF. The majority of journals had an IF that is lower than three, regardless of the volume of articles published for both countries. The majority of international journals from Europe or North America had IF that were higher than those from SA and BRA. The vast majority of journals had IF less than two, reflecting the importance of the number of publications cited produced as this influences the journal ranking status. However, Seglen, in "Why the impact factor of journals should not be used for evaluating research" [23], states that this could not be an accurate measure as IF conceals the variations of citation rates within the journal; that the IF is unable to measure the scientific quality of publications; that IFs are influenced by the research field and its rates of growth and development; and that the rate of article citation is the independent factor influencing the journal IF [22].

In terms of open access designation, the DOAJ is an independent community-curated online directory that indexes high quality and peer-reviewed journals. Other publications, related to both countries, had a green accepted and published designations, indicating that authors were free to archive a copy in a website or any repository that can be accessible for free for non-commercial use, however, the copyright is retained by the publisher or funder with restrictions on self-archiving. The green designation is inexpensive; but has an embargo period and may not include edits after peer-review, but is accessible thereafter at no extra charge [23].

The gold designation enabled the final version of the article to be free and permanently accessible to everyone, immediately after publication, with the copyright retained by the authors. However, this designation is costly and the article processing charge can be as high as $4000 US dollar. There were more publications with bronze designations indicating that authors can access articles from the publisher’s website and page but are restricted to reuse the papers and the journal may not have identifiable license [23].

## Limitations

The study limitations include articles indexed only in WoS. Other databases such as Scopus, PubMed, SciVal and Clintrials.gov and other databases such as social sciences and arts and humanities, which may have revealed different results or identified other trends, different from our results. In addition, including other databases may have provided a comprehensive volume of CRC publications, written in various languages, related to each country and promoted detailed analysis of relationships, reflected scope of research capacity that exists in each country and identified between county interactions excluded by WoS. Further analysis of links and interaction between cited reference counts and countries could have assisted in identifying further interactions and collaboration.

Focusing only on publication title, keywords and abstract to conduct content analysis, instead of reading full articles, we may have missed important information that may have allowed us to draw strong and definitive conclusions using the bibliometric analysis. It may have helped to also include a Social Network Analysis, to identify the prominent role of each country on the production of CRC publications. It would have also assisted to identify research areas determining the size and relevance of publications network.

Including publications covering all countries in the world or the region, and author with affiliation to the country of analysis, we may have made assumptions of applicability when there were none that existed. Not all the needed information was included such as funding sources, research organisations; open access designation as some of the records did not contain data in the field being analysed, to fully understand current CRC status in each country and interactions and the collaboration between the two countries.

## Conclusion

The importance of on-going bibliometric studies cannot be overemphasised, noting the pace and variation of research generation and development progress in the developing countries. To the author’s knowledge, this is the first paper that conducted bibliometric analysis on CRC publications, especially, related to SA and BRA, providing unique insights related to the CRC research focus and development in developing countries.

Reviewing publications between the years 2000 to 2020 showed a 20-year timespan adequate to show trends among research areas for publications related to both countries. Importantly, this analysis provides an important contribution in the synthesis of research on CRC as impetus to generate additional knowledge on CRC.

Given the rising contributions of individual BRICS countries to CRC knowledge generation, BRICS countries should consider allocating more domestic funds or through public-private partnerships to increase or establish new collaborations on CRC research activities within country, region and between BRICS countries to achieve the Cancer Control Goals.

The increased funding for CRC research may promote strong local scientific leadership, transparency high-quality data and increase CRC research capacity, knowledge, evidence-based learning and resource sharing, which are key elements for effective CRC cancer control.

The study also highlighted the importance of developing or strengthening the national CRC surveillance system to promote productivity, identify best practices and expand research categories with diverse number of authors generate the evidence that can influence policy and practice to control CRC burden. This study will inform policy, planning and funding decisions on CRC in each country and within BRICS.

## Authors’ contributions

Conceptualisation SM; methodology – SM, GVH; analysis, writing review and editing – SM, GVH, MH.

## Conflicts of interest

The authors declare that they have no conflicts of interest.

## Funding

There was no funding for this research.

## Figures and Tables

**Figure 5a.**
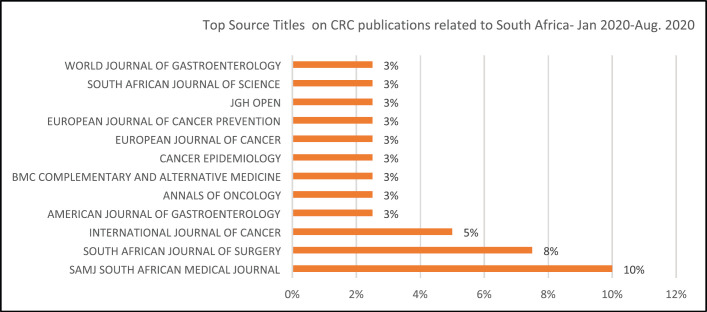
Top journals on CRC publications to SA: Jan. 2020–Aug. 2020.

**Figure 5b.**
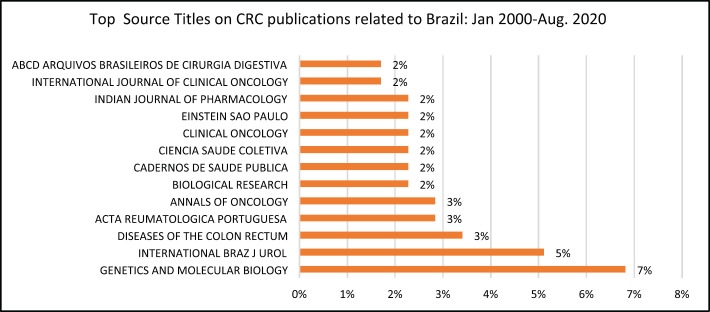
Top source titles on CRC publications related to BRA: Jan. 2020–Aug. 2020.

**Table 1.** The top four authors and number of publications per author on CRC related to SA and BRA: Jan. 2000–Aug. 2020.

Authors (SA)	Number of publications	% of publications (*n* = 80)	Authors (BRA)	Number of publications	% of publications (*n* = 176)
Ramesar R	8	10	Ashton-Prolla P	10	6
Algar U	5	6	Iwasaki M	8	5
Goldberg PA	5	6	Palmero EI	8	5
Ruff P	5	6	Sharma S	8	5
Katsidzira L	4	5	Tsugane S	8	5
Madiba TE	4	5	Coy CSR	7	4
Rusakaniko S	4	5	Le Marchand L	7	4
Thomson S	4	5	Lima CSP	6	3
Bebington B	3	3	Miyajima NT	6	3
Gangaidzo IT	3	3	Novaes MRCG	6	3
Matenga JA	3	3	Reis RM	6	3
Penny C	3	3	Rossi BM	6	3

**Table 2a.** The top six research organisations identified with CRC publications related to SA: Jan 2000–Aug. 2020.

Research organisations	Number in SA	% of 80 records
University of Witwatersrand (Wits)	21	26%
University of Cape town (UCT)	15	19%
University of Kwazulu Natal (UKZN)	8	10%
South African Medical Research Council	5	6%
National Health Laboratory Service	4	5%
University of Zimbabwe	4	5%
World Health Organization	4	5%
International Agency for Research on Cancer (IARC)	3	4%
University of Pretoria	3	4%
University of the Western Cape	3	4%

**Table 2b.** The top six research organisations identified in BRA with CRC related publications: Jan. 2000–Aug. 2020.

Research organisations	Number in BRA	% of 176 records
Universidade de Sao Paulo	40	23%
A C Camargo Cancer Center	17	10%
Hospital de Cancer de Barretos	14	8%
Universidade Federal do Rio Grande do Sul	14	8%
Fundacao Oswaldo Cruz	13	7%
Universidade Federal de Sao Paulo (UNIFESP)	11	6%
Universidade Estadual de Campinas	10	6%
Universidade Federal de Minas Gerais	9	5%
Universidade Federal do Rio de Janeiro	9	5%
Hospital Sirio Libanes	8	5%
National Cancer Institute (INCA)	8	5%

**Table 3.** Common journals that published CRC papers related to SA and BRA between Jan. 2000 and Aug. 2020.

Common document source titles	SA – of 80 records	BRA – of 180 records
International Journal of Cancer	5%	1%
Annals of Oncology	3%	3%
Cancer Epidemiology	3%	1%
European Journal of Cancer Prevention	3%	1%
World Journal of Gastroenterology	3%	1%
BMC Cancer	1%	1%
BMC Gastroenterology	1%	1%
Cancer	1%	1%
Cancer Medicine	1%	1%
Colorectal Disease	1%	1%
Diseases of the Colon Rectum	1%	3%
ecancermedicalscience	1%	1%
Lancet Gastroenterology and Hepatology	1%	1%
Lancet Oncology	1%	1%
Medical Hypotheses	1%	1%
Oncology Reports	1%	1%
PLOS Medicine	1%	1%
Public Health Nutrition	1%	1%
Scientific Reports	1%	1%
World Journal of Surgery	1%	1%

**Table 4.** Source, titles of publication with most cited reference count.

Country	Source titles	SA cited reference count (%)
SA	SAMJ South African Medical Journal	7%
	South African Journal of Surgery	5%
	Cancer and Metastasis Reviews	5%
	World Journal of Gastroenterology	4%
	Biofactors	4%
BRA	Biology	5%
	Nutricion Hospitalaria	5%
	Public Health Nutrition	5%
	Cadernos de Saude Publica	4%
	Food Research International	4%
	PLOS One	4%

**Table 5.** Most frequent journals that published CRC research related to SA and journal IF.

Journal titles (SA)	Number of records	% of 80 records	IF
SAMJ South African Medical Journal	8	10%	1.285
South African J. Surgery	6	8%	none
International Journal of Cancer	4	5%	**5.145**
European Review for Medical and Pharmacological Sciences	2	3%	3.024
Journal Global Health Open	2	3%	none
Annals of Oncology	2	3%	**18.274**
European Journal of Cancer Prevention	2	3%	2.413
BMC Complementary and Alternative Medicine	2	3%	2.833
European Journal of Cancer	2	3%	2.413
World Journal of Gastroenterology	2	3%	3.665
American Journal of Gastroenterology	2	3%	**10.171**
Cancer Epidemiology	2	3%	2.179
South African Journal of Science	2	3%	1.866

**Table 6.** Most frequent journals that published CRC research related to BRA and journal IF.

Journal titles (BRA)	Number of records	% of 176 records	IF
Value in Health	12	6.818	1.736
Annals of Oncology	9	5.114	**14.196**
Familial Cancer	6	3.409	1.015
Cadernos de Saude Publica	5	2.841	1.15
Journal of Clinical Oncology	5	2.841	**14.314**
Diseases of the Colon Rectum	4	2.273	**3.556**
Genetics and Molecular Biology	4	2.273	1.271
Nutricion Hospitalaria	4	2.273	0.951
PLOS One	4	2.273	2.806
Public Health Nutrition	4	2.273	2.284
World Journal of Gastroenterology	4	2.273	2.369
BMC Cancer	3	1.705	3.15
Genetics and Molecular Research	3	1.705	1.271
International Journal of Colorectal Disease	3	1.705	2.710
Molecular Biology Reports	3	1.705	1.889
Revista Panamericana de Salud Publica Pan American Journal of Public Health	3	1.705	1.309
